# Lithotrity and Lithotomy

**Published:** 1830-01-01

**Authors:** 


### XXXVI.

### LITHOTRITY AND LITHOTOMY.

Every body knows, or at all events ought to know, that now and then the public requires something new to tickle its jaded appetite, and enable it to get through its ordinary fare without nausea and indigestion. What in the world could the Londoners do in the autumnal months, if they had not a fire-eater or the Grand Turk and Count Diebitsch to astonish and confound them? They would die in their smoke to a certainty. Like our neighbours we (that is to say doctors) must have our nine days' wonder, and so that it is strange and incredible, it does not much signify what the matter may be. The all engrossing subject, the *magnifique chose* of the present day, is lithotrity beyond a question. The professors of this great discovery are at present most busily occupied in *boring* their patients, the public, and each other on both sides of the Straits of Dover. This is as it should be, and provided the gentlemen themselves are satisfied, most assuredly we have no reason to be otherwise.

Whenever any thing new is promulgated, whether it be a patent corkscrew or a scientific principle, two circumstances are always observed. First, the corkscrew or the principle, be it which it may, is announced as infinitely superior to all other corkscrews or principles; and secondly a host of claimants start up and assert that it is no discovery at all, but that *they* have known the identical thing for God knows how many years. This mode of proceeding is so constant on these occasions that we are not a whit surprised at remarking it at present in the case of lithotrity. The enthusiasts for the operation, not content with the merits it really possesses, must exalt it above any thing short of the philosopher's stone, and one of these visionaries asserted in a thesis which he sustained at Paris—"that if a portion of the mucous membrane of the bladder were seized by the instrument and torn away, it would be a very desirable and fortunate circumstance, because of the local bleeding which would follow."! M. Civiale even maintains that lithotrity, so far from being likely to injure the bladder, is on the contrary calculated to improve its condition when diseased, (détérioré)! Such opinions as these requiring to be taken cum grano salis, it would be a very desirable thing to obtain an impartial estimate of the advantages and disadvantages of the operation. But where shall we get that unbiassed judgment? There's the rub. We have seen, and we might know it if we had not seen it, that the discoverers or adepts are not unprejudiced, and those who are neither discoverers nor adepts cannot of course form an accurate opinion. The only real arbitrator in these matters is Time, which determines pretty fairly the value of all things.

But as Time is like the Chancellor a tardy sort of judge, who won't suit your peppery disputants of 1829, it becomes necessary to anticipate the decision as far as one can, even at the risk of having the rule discharged at last with costs. Be not alarmed Messrs. Leroy d'Etiolle, Civiale, and Heurteloup, *we* are not about to decide upon your claims, nor to trim the balance between lithrotrity and lithotomy. We know too well—

> The dangers that environ
> The man who meddles with cold iron, to settle the merits of the gorget and the pince à trois branches. We merely intend to glance at a work lately published in Paris by a M. Bancal, a physician of Bordeaux, and a lithotritist. It is entitled a "Practical Manual of this Operation," and is written in the form of letters to a young physician. Our object is not to analyse the work but merely to extract one or two passages from it, in order to place the actual bearings of the question on something like an approximation to the truth. A word or two may be said with regard to the introduction of the straight instrument into the bladder. M. Bancal divides it into three stages;—in the first, the end of the instrument is carried as far as the bulb, the penis being held vertically; in the second, the penis and the instrument are slightly inclined downwards, and the beak of the latter traverses the membranous part of the urethra; in the third, the instrument and penis are depressed still more towards the thighs, and the blunt point of the former slips through the prostatic portion of the canal, mounting over the eminence formed by the third lobe of the prostate gland. So much for the introduction of the straight instrument, and we have no intention of detailing the steps of the process of drilling, as so many descriptions of that are already before the profession.

The tenth letter is occupied with the question of lithrotrity for calculi in the female bladder, and a very practical one it is. M. Bancal has tried it in three cases, and experienced the *greatest difficulty* in each, a difficulty "which has also been felt by other persons." We have reason to know that such is the fact, and refer our readers to a case related by Mr. James Wilson of Glasgow, and noticed in the Periscope of the present number. M. Bancal speculates on the why and wherefore, but without any great success. He imagines that the principal cause is the low situation of the neck of the bladder, at its very *bas-fond*, in consequence of which the stone always lodges on the part, and is applied on the opening into the urethra. This is ingenious, but, as a contemporary French critic well observes, the elevation of the pelvis ought to obviate such a difficulty, which however it does not. The critic to whom we allude, a writer in the Hebdomadaire evidently well acquainted with his subject, proposes another conjecture: viz. that the great transverse extent of the female bladder, and the depression of its *bas-fond* on the sides of the vagina are the real obstacles to the seizure of a stone. We are inclined to think that this idea is correct, and that the true solution of the problem will be found in the great relative capacity of the organ, which capacity must, of course, be chiefly in the transverse diameter. Whatever may be the cause it is certain that lithotrity is not well adapted for the female, and the present operation on that sex is not only more easily but more expeditiously performed. This is the important point, and all parties appear to be agreed upon it.

The last and most complex question that remains is the comparative merit of lithotrity and lithotomy in the male. It is very clear that to settle this point we are not to take for granted the assertions of the ultra advocates of either operation. Raw, for instance, as arrant a knave as ever juggled at a country fair, asserts that he cut 1500 persons for the stone, without having lost a single individual. On the other hand, M. Bancal declares in his preface that lithotrity is of no more consequence than the introduction of a catheter, and says in express terms that it is "always innocent." We shall see in a few minutes the innocence of his pet. When we set aside these extravagant pretensions, we have still another impediment to encounter in arriving at the truth. The lithotritists have notoriously *picked* their cases; that is, they have selected for their operation persons affected with small single stones, and free from any other disease of the bladder, kidney or urethra; persons in whom, as the intelligent writer in the Hebdomadaire observes, the lateral operation of lithotomy would have offered the greatest chance of success. We know that this has been in some degree denied, but out of their own mouths will we judge the lithotritists :—"Laissons aux lithotomistes," says M. Bancal in his sixth letter, "*le soin de porter l'instrument tranchant dans les vessies malades.*" This good natured offer will no doubt be duly appreciated by the public and the lithotomistes. Under these circumstances it is obvious how impossible it is at present to decide on the substantive value of lithotrity, but the following table of calculous patients who presented themselves to M. Bancal, will perhaps dispel some of those airy castles erected in the brains of our cockney doctors, respecting the universal "innocence" of the operation.

Eleven patients with stone applied to M. Bancal. Of these, five were subjected to the operation of lithotrity.

Two of the five were cured :—M. Angault and M. Dupuis. The other three died of inflammation of the bladder; — two after the operation: — L'Eglise and Eusebi, a Spaniard; one during the process of dilating the urethra preparatory to lithrotrity :—M. Figuier.

Two patients supported some attempts at lithotrity, and then would submit to it no longer :—M. S. and M. Orignac.

The remaining four of the eleven were *lithotomized* with success;—one by M. Viguerie of Thoulouse (M. Rollan);—three by M. Bancal himself;—M. Delerm, M. B. Hugot, and Anna, a sailor, in whom the stone was sacculated and could not be extracted.

The foregoing table is any thing but favourable to lithotrity, two only out of seven in whom it was performed, being cured;—three dying of inflammation of the bladder;—and two refusing to submit to its performance, after one or two trials. We cannot but praise the candour with which the results are thus made public by M. Bancal, and we sincerely wish that every medical writer would evince the same. We should not then be inundated with the lies (why mince the matter?) that mislead us at every step in physic. We do not put forward these unfavourable cases as shewing the actual value of the new operation, on the contrary, we are disposed to take its part against the operator, M. Bancal, and consider them as much more unfortunate than usual. At the same time it is an act of justice, and, on our parts an act of duty, to make public such facts of importance as these, and destroy the ridiculous impression on the part of many, that no danger whatever can possibly attach to lithotrity.

